# Identification of Mutated Peptides in Bladder Cancer From Exomic Sequencing Data Reveals Negative Correlation Between Mutation-Specific Immunoreactivity and Inflammation

**DOI:** 10.3389/fimmu.2020.576603

**Published:** 2020-11-30

**Authors:** Chen Wang, Yu Ding, Yuanyong Liu, Qingchen Zhang, Shiqiang Xu, Liliang Xia, Huangqi Duan, Shujun Wang, Ping Ji, Weiren Huang, Guoping Zhao, Zhiwei Cao, Haibo Shen, Ying Wang

**Affiliations:** ^1^ Department of Urology, Xinhua Hospital, Shanghai Jiaotong University School of Medicine, Shanghai, China; ^2^ Department of Immunology and Microbiology, Shanghai Institute of Immunology, Shanghai Jiaotong University School of Medicine, Shanghai, China; ^3^ Deparment of Gastroenterology, Shanghai Tenth People’s Hospital, School of Life Sciences and Technology, Tongji University, Shanghai, China; ^4^ State Engineering Laboratory of Medical Key Technologies Application of Synthetic Biology, Shenzhen Second People’s Hospital, The First Affiliated Hospital of Shenzhen University, Shenzhen, China; ^5^ Shanghai-MOST Key Laboratory of Health and Disease Genomics, Chinese National Human Genome Center at Shanghai, Shanghai, China

**Keywords:** bladder cancer, the Cancer Genome Atlas database, mutated peptides, immunoreactivity, inflammation, neoantigen

## Abstract

**Introduction and Objective:**

Neoantigen-based immunotherapy is one of the breakthroughs in cancer immunotherapy. Benefit from the Cancer Genome Atlas database, we intended to identify mutant peptides with neoantigen property in bladder cancer (BC). Correlations between the immunoreactivity of candidate neoantigens and clinical manifestations were further analyzed.

**Methods:**

HLA-A*02:01 restricted mutant (MT) and wildtype (WT) peptides were predicted by using whole exome sequencing data of 412 BC patients in the TCGA database. Binding affinity to HLA-A2 molecules was determined by using T2 cell-based binding assay. The immunoreactivity to WT and MT peptides in HLA-A2^+^ BC patients was determined by using an ELISPOT assay upon *in vitro* stimulation with MT and WT peptides individually. Clinical relevance to peptide-specific immunoreactivity was analyzed by Pearson correlation analysis. The disease free survival (DFS) curves were plotted using the Kaplan–Meier method in BC patients with or without mutations and compared using the log-rank test online.

**Results:**

Fifty-seven HLA-A*02:01 restricted WT and MT peptides were selected based on predicted high affinity and expression frequency, among which 12 MT peptides from 12 individual genes exhibited strong affinity to HLA-A2 molecules when compared to WT counterparts. MT peptides induced more peptide-specific IFNγ spot forming units (SFUs) than WT counterparts in HLA-A2^+^ BC patients upon *in vitro* stimulation. They were negatively correlated to the counts of peripheral leukocytes and platelets. Patients with higher C-reactive protein level exhibited lower immunoreactivity to MT peptides. Combination of MT peptides from 6 genes, including *CDKN1A^G61V^*, *RHOB^P75L^*, *DDB1^S25L^*, *AHNAK^D4855Y^*, *ANP32A^S56L^* and *MKI67^H84L^* covered 47.5% of the patients under investigation. Patients harboring combinational mutations in these genes were associated with a longer DFS according to the cBioportal online analysis.

**Conclusion:**

Twelve HLA-A*02:01 restricted MT peptides have been identified exhibiting higher binding affinity to HLA-A2 molecules and stronger immunoreactivity than WT counterparts in BC patients. Combination of MT peptides from six genes might be potential as neoantigen candidates in cancer immunotherapy against BC in the future. Inflammatory modulation is inclined to be a strategy to enhance the efficacy of neoantigen-based immunotherapy.

## Introduction

Bladder cancer (BC) originates from transitional cells of bladder urothelium. It is the sixth most common cancer in Europe and the United States (1, 2). In China it is estimated with 80,500 new cases and 32,900 deaths of BC in 2015 (3). The vast majority of patients are diagnosed as non-muscular invasive bladder cancer (NMIBC) and receive transurethral bladder tumor resection. At present, the 5-year recurrence rate of NMIBC is 31–78%. Up to 45% of the patients at the first follow-up cystoscopy have suffered recurrence, and 6–17% of them progress into muscle invasive bladder cancer in the long-term follow-up (4). The etiology and pathophysiology of BC are not well addressed yet. New strategies to improve the clinical diagnosis and the treatment of BC are thus still challenging.

Besides the surgery and chemotherapy, intravesical administration of Bacille Calmette-Guérin (BCG) is one of the most successful examples of immunotherapy through inducing regional and systemic anti-tumor immunity (5, 6). In fact, cancer immunotherapy blooms in the last several years due to the great success in immune checkpoint inhibitor treatment for multiple malignancies (7). Neoantigen-based immunotherapy raising great interests lies in the proof-of-concept investigations on the existence of somatic mutations in malignant cells based on next-generation sequencing data. Whole exome sequencing (WES) oriented data mining of mutated peptides not only drafts the mutational landscapes of tumor cells (8, 9), but also provides new tools both for the prediction of patient survival (10) and the selection of the peptides as candidate vaccines against tumors in the mice and human (11).

Owing to the implementation of the Cancer Genome Atlas project (TCGA) and the advance in bioinformatics pipeline, BC has been estimated with a mutation load above 10 somatic mutations per megabase of coding DNA comparable to lung cancer and colorectal cancer (12). Considering the availability of T cell recognition for neoantignes in melanoma with a similar mutation load (13, 14), somatic mutation profiles and neoantigen repertoires in BC is worthy of in-depth investigation. What is more, whether somatic mutated peptides can trigger peptide-specific T cell reactivity in BC patients is more significant. This might help to decipher the relationship between somatic mutations and clinical outcomes as well as the development of vaccine-based immunotherapy.

In the present study, based on the WES data from 412 BCs in the TCGA database we intended to identify the existence of mutant peptides with neoantigen property in BC. The immunoreactivity to candidate neoantigens was evaluated in BC patients. Correlations between the immunoreactivity to candidate neoantigens and clinical manifestations were further analyzed. This will facilitate the applications of neoantigen-based immunotherapy in BC.

## Materials and Methods

### Patients and Blood Collection

One hundred forty BC patients receiving the surgery in the Department of Urology, Xinhua Hospital affiliated to Shanghai Jiaotong University School of Medicine between Sep 2017 and June 2019 were recruited in the study. The inclusion criteria were: (1) No immunosuppressant in use; (2) histologically confirmed urothelial carcinoma; (3) HLA-A2^+^. The exclusion criteria were: (1) BC patients receiving preoperative chemotherapy/radiotherapy; (2) BC patients had hematological or autoimmune diseases; (3) Lack of detailed clinical information and laboratory information. Accordingly, 100 patients were excluded. Among them, 82 patients were HLA-A2 negative. Four patients had hematological or autoimmune diseases. Six patients had incomplete medical data and 8 patients had incomplete laboratory data. Finally, a total of 40 patients were enrolled in the study. The clinical–pathological characteristics of 40 HLA-A2^+^BC patients were summarized in **Table 1**. This study was approved by the Medical Ethics Committee of Xinhua Hospital affiliated to Shanghai Jiaotong University School of Medicine. All the patients enrolled have signed informed consent forms. All the procedures were conducted in accordance with the Declaration of Helsinki. Before the surgery, 20 mL whole blood was collected in EDTA-containing tubes for further experiments.

**Table 1:** Demographic descriptions.

Characteristics	Cohort (n=40)
**Mean age ± SD (years)**	68 ± 13
**Gender**	
male	33(82.5%)
female	7 (17.5%)
**Pathology**	
Pathologic Tumor Stage	
Ta, T1	32 (80%)
T2	4 (10%)
T3	3 (7.5%)
T4	1 (2.5%)
Pathologic Nodal Stage	
N0	40(100%)
Histology	
Urothelial carcinoma	40 (100%)
**Inflammatory Index**	
Median leukocyte, 10^9/L(IQR)	6.35(4.93–8.73)
Median neutrophils, 10^9/L(IQR)	3.77(2.83–6.39)
Median neutrophils proportion, %(IQR)	61.7(55.65–73.35)
Mean ± SD lymphocytes, 10^9/L	1.59 ± 0.54
Mean ± SD lymphocytes proportion, %	24.91 ± 11.03
Median monocytes, 10^9/L(IQR)	0.50(0.40-0.70)
Mean ± SD monocytes proportion, %	7.95 ± 2.39
Median platelets, 10^9/L(IQR)	180.50(153.25–225.25)
Median neutrophil/lymphocyte ratio (IQR)	2.39(1.67–4.99)
Median platelet/lymphocyte ratio (IQR)	114.27(89.76–184.72)
Mean ± SD lymphocyte/monocyte ratio	3.27 ± 1.56

IQR, interquartile range.

### Data Source

The WES data of 412 BC tumor and paired peripheral neutrophils were downloaded from the Broad Institute Genome Data Analysis Center (https://gdac.broadinstitute.org/). Protein sequences of transcripts were downloaded from the National Center for Biotechnology Information (NCBI) (https://www.ncbi.nlm.nih.gov/).

### Bioinformatics Workflow for Wild Type and Mutant Peptide Prediction

By mapping to reference sequences in NCBI, a total of 84719 non-synonymous somatic mutations were obtained in 412 BCs. Their transcripts were converted to protein sequences. The sliding window of 9 amino acids was used to generate all possible mutant (MT) peptides covering mutation positions. Corresponding peptides were generated from blood data as wild-type (WT) counterparts. All MT and WT peptides were subjected to the affinity prediction to HLA-A*02:01 by using online NetMHCpan software (http://www.cbs.dtu.dk/services/NetMHCpan-3.0/). MT peptides with strong binding (affinity<500) and their WT counterparts with weak binding (affinity>500) were ranked (**Table 2**, list 1). Meanwhile, their relative expression levels were ranked by RNAseq data (**Table 2**, list 2). R package of RankAggreg was used to aggregate the two rank lists into the final score. The top ranked peptides with high scores were those with strong binding affinity to HLA-A*02:01 and high RNA expression level while WT counterparts had weak binding to HLA-A*02:01. The top 57 peptides were synthesized in Shanghai Sangon Biotech (Shanghai, China) and purified through high performance liquid chromatography by the company. They were hydrophilic and were dissolved in phosphate buffer saline at the concentration of 8mg/mL for the storage at −80°C.

**Table 2:** The information of 57 predicted HLA-A*02:01 restricted wildtype (WT) and mutant (MT) peptide pairs.

Gene	Substitution (WT,AA#,MT)	Predicted peptides				Expression	Score	Gene	Substitution (WT,AA#,MT)	Predicted peptides				Expression	Score
		WT	Affinity	MT	Affinity					WT	Affinity	MT	Affinity		
AHNAK	D4855Y	**D**LDLKGPKV	10911.65	**Y**LDLKGPKV	21.03	19116.15	208186680.1	EML4	G786V	QVFGVWPE**G**	19946.11	QVFGVWPE**V**	31.08	1711.52	34084938.73
PPIB	D56Y	**D**VGRVIFGL	13532.17	**Y**VGRVIFGL	41.73	13270.20	179020773.9	DPP9	R854L	I**R**AGKPYQL	18819	I**L**AGKPYQL	21.79	1810.09	34024615.98
ATF4	D336Y	**D**LIEEVRKA	17143.93	**Y**LIEEVRKA	22.83	6800.98	116440272.2	CC2D1A	P813L	V**P**AAVPTQV	20362.16	V**L**AAVPTQV	17.41	1616.66	32890601.35
MYH9	E1347K	**E**MIQLQEEL	4348.57	**K**MIQLQEEL	46.01	25557.15	109961178.7	RUNX1	R130I	A**R**FNDLRFV	16628.02	A**I**FNDLRFV	20.36	1926.20	31989630.5
CAP1	K37Q	S**K**AGAAPYV	11105.08	S**Q**AGAAPYV	13.72	8707.71	96580330.6	FNDC3B	S1042L	F**S**ETYTFST	13947.8	F**L**ETYTFST	26.58	2285.61	31818437.06
SYNPO2	P652L	Q**P**APWSQPA	26326.41	Q**L**APWSQPA	40.51	3512.41	92326931.54	TP63	S189L	K**S**ATWTYST	9726.04	K**L**ATWTYST	15.67	3218.87	31256418.99
RHOB	P75L	Y**P**DTDVILM	16245.84	Y**L**DTDVILM	15.23	5673.98	92092093.75	LIG1	P283L	K**P**GQKVPYL	22825.32	K**L**GQKVPYL	22.26	1317.29	30038344.31
SND1	R466L	GLATVIRY**R**	20843.64	GLATVIRY**L**	29.34	4335.36	90237563.24	ANP32A	S56L	T**S**IANLPKL	9377.99	T**L**IANLPKL	22.14	3112.45	29119655.22
CDKN1A	G61V	FVTETPLE**G**	14683.37	FVTETPLE**V**	24.21	5955.84	87307609.97	COL12A1	S395L	L**S**ADTEYQI	6121.63	L**L**ADTEYQI	12.3	4560.59	27862146.59
ACTB	R196C	YLMKILTE**R**	674.92	YLMKILTE**C**	18.33	122917.87	80706643.61	ALDH16A1	G343V	GLDGAVDM**G**	18989.79	GLDGAVDM**V**	41.31	1376.70	26086296.66
PPP2R1A	S314L	L**S**ADCRENV	9585.42	L**L**ADCRENV	19.49	8184.12	78288742.91	DSG3	S273L	Y**S**ARIEENI	9423.05	Y**L**ARIEENI	44.34	2684.78	25179791.6
ITGB4	S344L	HLLDSKVP**S**	5661.01	HLLDSKVP**L**	40.66	12521.32	70374208.67	MET	R1148Q	L**R**SEGSPLV	12216.93	L**Q**SEGSPLV	13.19	2054.12	25067898.47
MKI67	H84L	K**H**GDVITII	23696.01	K**L**GDVITII	27.55	2799.86	66268274.96	ATP13A1	R1079L	YLYREAQA**R**	11916.14	YLYREAQA**L**	15.58	2105.82	25060418.29
PRDX1	F42L	F**F**FYPLDFT	5854.1	F**L**FYPLDFT	17.61	10921.26946	63741879.99	CD81	P53S	K**P**APNTFYV	2417.19	K**S**APNTFYV	48.97	10442.95	24731207.5
DDB1	S25L	T**S**AEDLNLL	10431.97	T**L**AEDLNLL	14.88	6098.820878	63531965.98	PIK3C2B	R843Q	K**R**YYCHSEV	15638.41	K**Q**YYCHSEV	45.87	1584.98	24713905.56
AZIN1	G376V	CLLPELNV**G**	12266.33	CLLPELNV**V**	29.87	3945.14276	48274581.58	TAOK3	R676L	I**R**LQHQTEL	26267.8	I**L**LQHQTEL	39.49	930.91	24416274.22
TP53	R273L	F**R**LGFLHSG	26951.83	F**L**LGFLHSG	35.31	1698.218142	45710122.58	RHOB	S85L	F**S**VDSPDSL	4088.4	F**L**VDSPDSL	10.19	5673.98	23139666.2
SIK1	P84L	QLMKLLNH**P**	9706.8	QLMKLLNH**L**	37.74	4408.544858	42626484.74	ADIPOR1	G328V	RIPERFFP**G**	8412.46	RIPERFFP**V**	11.26	2752.04	23120429.97
RUVBL1	P412L	T**P**ANLLAKI	26279.17	T**L**ANLLAKI	49.33	1612.110236	42285393.55	PRDX6	R108W	IIDDRN**R**EL	4439.42	IIDDRN**W**EL	34.63	5202.54	22916095.26
IRF2BP2	S195L	G**S**ATPLPTA	11596.13	G**L**ATPLPTA	45.06	3548.308467	40986759.48	LRRC14	P27L	L**P**RELFPLL	24049.1	L**L**RELFPLL	49.58	941.28	22590297.36
CDC42	P73L	Y**P**QTDVFLV	5996.74	Y**L**QTDVFLV	2.89	6753.099448	40477065.13	MFSD5	R280Q	A**R**AAFWNHV	18110.07	A**Q**AAFWNHV	43.27	1246.80	22525601.83
HK2	E774V	LLFRGRIS**E**	16022.92	LLFRGRIS**V**	24.04	2502.109804	40030954.5	GAK	F529L	L**F**STAEAAV	9737.94	L**L**STAEAAV	35.72	2306.14	22374682.88
CREBBP	R1446L	L**R**TAVYHEI	22971.74	L**L**TAVYHEI	26.32	1721.651278	39504011.67	MYO5B	D1586Y	**D**LTEYRQVL	15279.98	**Y**LTEYRQVL	25.45	1446.60	22067162.35
SUMO1	P58L	V**P**MNSLRFL	18628.77	V**L**MNSLRFL	19.41	2091.24	38916550.19	DNAJC13	F1325L	L**F**SKESPLL	16080.23	L**L**SKESPLL	42.47	1366.18	21910499.3
TNKS2	P804L	L**P**SCYKPQV	25549.37	L**L**SCYKPQV	19.97	1498.065	38244701.73	PCBP1	S223L	Y**S**IQGQHTI	2607.71	Y**L**IQGQHTI	8.79	8300.84	21573226.87
EIF4G2	F855L	MLLRFFVH**F**	2067.69	MLLRFFVH**L**	16.69	18414.53	37768196.89	NUP210	S194L	S**S**ANSILHI	10602.55	S**L**ANSILHI	39.96	2034.74	21492140.04
RUNX1	R205L	RLSELEQL**R**	19337.71	RLSELEQL**L**	24.63	1926.20	37200803.3	GAK	S829L	V**S**DEGGSPI	9240.21	V**L**DEGGSPI	42.09	2306.14	21212157.43
CDK16	S92L	A**S**ATSSDEV	12127.5	A**L**ATSSDEV	40.76	2835.29	34269380.71	PNPLA2	F147L	G**F**IPVYCGL	8704.87	G**L**IPVYCGL	20.63	2399.23	20835460.71
OGDH	D612Y	**D**ILTHIGNV	11605.41	**Y**ILTHIGNV	21.58	2957.79	34262533.63								

*red colored letters: amino acid substitution.

### *In Vitro* Binding Affinity Assay

T2 cells were maintained in RPMI-IMDM medium containing 10% fetal bovine serum (FBS) (GIBCO, Grand Island, NY, USA) routinely in the lab. T2 cell-based *in vitro* binding affinity assay was performed as reported previously (15). Briefly, T2 cells (0.5 x 10^6^/mL) was incubated with β2 microglobulin (3 μg/mL) (Sigma-Aldrich, St. Louis, MO,USA) and individual peptide (10 μg/mL) for 4 h at 37°C. L235 peptide was used as a positive control while RPMI-IMDM medium (GIBCO) as a negative control (15). After the incubation, flow cytometric analysis was performed to determine the expression of HLA-A2 on T2 cells. Briefly, cells were incubated with PE-mouse anti human-HLA-A2 antibody (Clone BB7.2) (Sigma-Aldrich) for 40 min at 4°C and acquired by FACS Canto II (BD Biosciences, Franklin Lakes, NJ, USA). Data analysis was performed by using FlowJo software (Tree Star Inc., Ashland, OR, USA). Mean fluorescence index (MFI) was used to determine the expression level of HLA-A2 molecules on T2 cells. To determine the dissociation constant (Kd) value of each peptide, T2 cells were incubated with β2 microglobulin (3 μg/mL) and the peptides at different concentrations (0, 0.4, 2, 10, 20 μg/mL) followed by flow cytometric assay. Peptide concentration at 50% of maximum MFI represented the Kd values of each peptide.

### Enzyme-Linked ImmunoSpot (ELISPOT) Assay

Peptide-specific IFNγ release was determined by using an ELISPOT assay according to the manufacturer’s instruction (U-CyTech, Utrecht, Netherlands). Briefly, peripheral blood mononuclear cells (PBMCs) were isolated from freshly collected whole blood of BC patients by density gradient centrifugation using LymphoprepTM reagent (Axis-shield, Oslo, Norway). Ninety-six-well PVDF plates (Millipore) were coated with anti-human or mouse IFN-γ coating antibody overnight at 4°C. PBMCs (0.25×10^6^ cells/well) were added in the plate and stimulated with individual peptide (2 μg/mL) in RPMI-1640 culture medium containing 10% FBS, 100 U/mL penicillin, and 100 µg/mL streptomycin (all from GIBCO) for 20 h at 37°C. The secreting IFN-γ was detected by biotin-labeled detection antibody and horseradish peroxidase (HRP) conjugated streptavidin. Coloration was developed with AEC substrate solution for 30 min at room temperature in the dark. The reaction was stopped by thoroughly rinsing the PVDF membrane with demineralized water. The plates were air-dried and the spots were counted by an ELISPOT reader (BioReader Model 4000; Bio-Sys GmbH, Karben, Germany). The number of peptide-specific IFN-γ producing cells was calculated as spot-forming units (SFUs) per 2.5×10^5^ cells of individual peptide minus that of blank control.

### Statistical Analysis

Statistical analysis was performed using SPSS 21 software (IBM SPSS Software, USA). The correlations between peptide-specific SFUs and clinicopathological parameters were analyzed by using Pearson correlation analysis and Student t test. The disease free survival (DFS) originated from the TCGA 412 bladder cancer database and the DFS curves were plotted *in silicon* based on the cBio Cancer Genomics Portal (http://www.cbioportal.org) that was developed at Memorial Sloan-Kettering Cancer Center (MSKCC) (16–18). The dissociation constant (Kd) value was calculated by Graphpad Prism version 6.0 (Graphpad software Inc., CA, USA). *P*< 0.05 was considered as statistically significant.

## Results

### Prediction of HLA-A*02:01 Restricted WT and MT Peptides From TCGA Whole Exsome Sequencing Data of Bladder Cancer

Eighty-four thousand seven hundred nineteen mutations from 412 BC patients were downloaded from Broad Institute Genome Data Analysis Center, among which 64,134 mutations were non-synonymous corresponding to13,718 mutated protein sequences. They were subjected to online HLA mapping to 34 HLA alleles prevalent in Chinese population. Eight thousand eight hundred twenty-seven peptides restricted to 34 HLA alleles were predicted. Among them, 920 peptides restricted to HLA-A*02:01 (rank list 1) were selected to analyze their expression levels using RNA-seq data (rank list 2). The final scores of 920 peptides were calculated and the top 57 pairs of WT and MT peptides with HLA-A*02:01 restriction were selected for peptides synthesis. The information of peptide pairs selected, including gene name, mutation site, amino acid sequence, predicted affinity score, gene expression score and the final ranking score, was listed in **Table 2**. All gene mutation types were missense mutation.

### Determination of Binding Affinity to HLA-A2 Molecule

**Figure 1:**
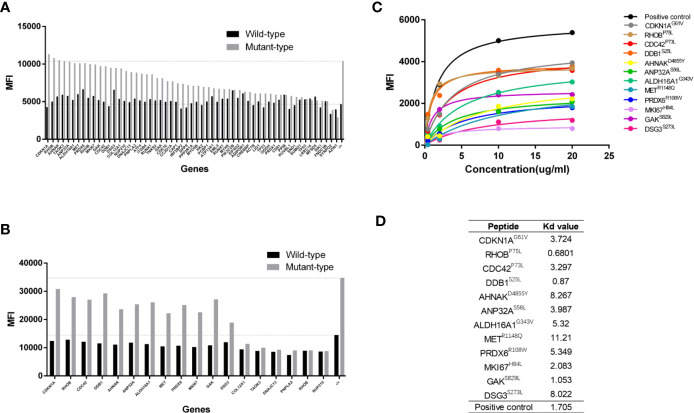
Determination of peptide binding affinity to HLA-A2. **(A)** The mean fluorescence index (MFI) values of HLA-A2 molecules on T2 cells after incubating with 57 pairs HLA-A*0201 restricted wildtype (WT) and mutant (MT) peptides individually. **(B)** The MFI values of HLA-A2 molecules from 18 pairs HLA-A*0201 WT and MT peptides in a repetitive experiment. **(C)** The MFI values of HLA-A2 molecules at different concentrations of 12 MT peptides. **(D)** The Kd values of 12 MT peptides.

The top 57 paired HLA-A*02:01-restricted MT and WT peptides were synthesized and subjected to an *in vitro* binding affinity assay using T2 cell. Results from the first set experiment showed that the mean fluorescence indexes (MFIs) of MT peptides were higher than or comparable to those of WT peptides, which is largely consistent with bioinformatics prediction (**Figure 1A**). We further selected 18 paired peptides whose value of the MFI was 1.5-fold higher in MT peptide than that in WT counterpart for further validation. Higher affinity to HLA-A2 molecules on T2 cells was confirmed in 12 MT peptides when compared to WT peptides (**Figure 1B**).

The Kd value of each peptide was further determined by using a series of peptide concentrations when incubating with T2 cells. It was obvious that the MFI values of MT peptides increased along with the elevated peptide concentrations and reached the plateau at 20 µg/mL (**Figure 1C**) whereas the MFIs of WT peptides did not increased dramatically (**Supplementary Figure 1**). The Kd values of MT peptides were within the range of 0.6–11 µg/mL with the highest affinity at 0.6801µg/mL (RHOB^P75L^) and the lowest at 11.21 µg/mL (MET^R1148Q^) (**Figure 1D**). However, there was no correlation between the Kd value and the predicted affinity of MT peptides (data not shown).

### Determination of the Immunoreactivity to WT and MT Peptides in the Periphery of BC Patients

**Figure 2:**
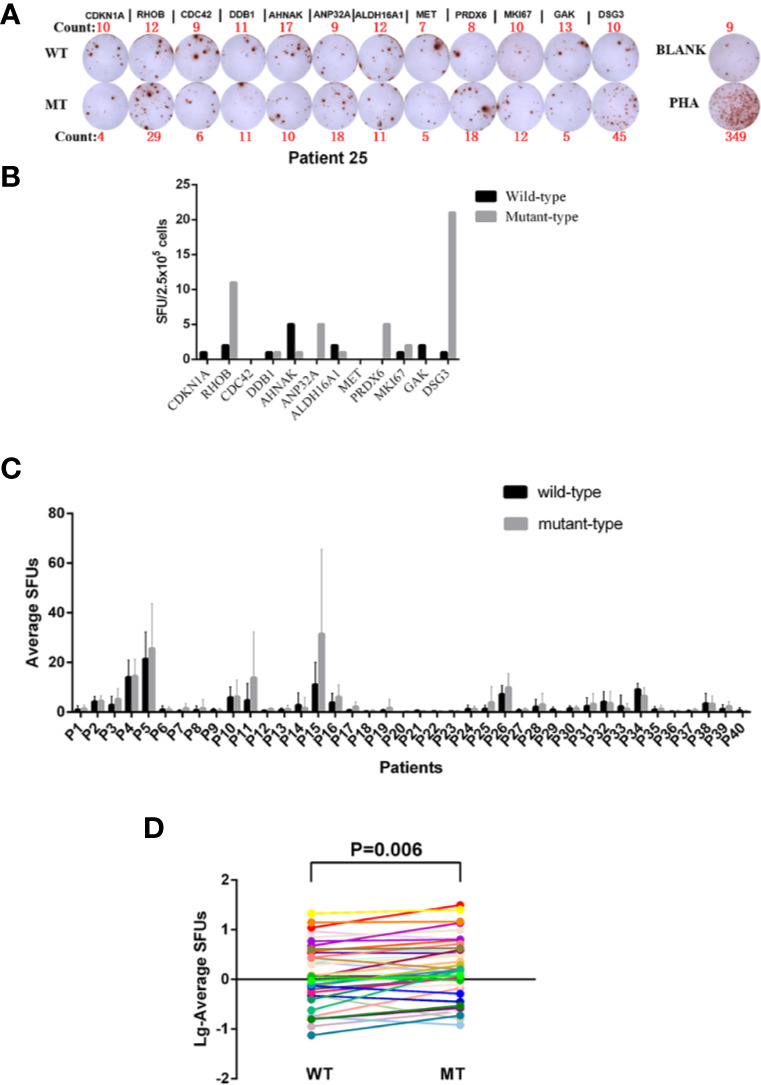
Determination of the Immunoreactivity to WT and MT peptides in HLA-A2^+^ BC patients. **(A)** Representatives of peptide-specific IFN-γ releases by an ELISPOT assay. PBMCs from Patient 25 were isolated and stimulated with 12 WT and MT peptides individually *in vitro* for 20 h. RPMI 1640 medium was used as BLANK while PHA as a positive control. The number of the spot-forming units (SFUs) of each well was shown as well. **(B)** The SFUs specific to 12 WT and MT peptides in Patient 25. **(C)** The average SFUs of 12 WT and MT peptides in 40 HLA-A2^+^ BC patients (the average SFUs = total SFUs from the tested peptides/the number of tested peptides). **(D)** Comparison of average SFUs between WT and MT peptides by the paired Student t test. Each line represented one patient.

Forty HLA-A2^+^ BC patients (**Table 1**) were recruited to determine the immunoreactivity to 12 paired WT and MT peptides in the periphery by an ELISPOT assay (**Figure 2A**). PBMCs from each patient were stimulated with 12 WT and MT peptides individually and peptide-specific IFNγ SFUs were counted (**Figure 2B** and **Supplementary Table 1**). The average SFUs upon *in vivo* stimulation of 12 WT and MT peptides were compared in 40 BC patients (**Figure 2C**). It was shown that MT peptides displayed higher immunoreactivity than WT peptides with elevated average SFUs in 40 BC patients (P = 0.006) (**Figure 2D**). These results indicate that consistent with high affinity to HLA-A2, MT peptides induce stronger immunoreactivity than WT in the periphery of BC patients. Nevertheless, there is no correlation between the average SFUs of 12 MT peptides and the relevant Kd value either (data not shown, P = 0.399). One healthy donor has been added to the screening just for comparison which exhibited few immune-reactivity to peptide stimulation *in vitro* (**Supplementary Figure 2**). Considering that stimulating the PBMCs from the patients only for 20 h maybe not the best condition, we have preformed the ELISPOT assays by stimulation of WT and MT peptides for 20 h and 60 h, respectively. The SFUs were similar between two time points (**Supplementary Figure 3**).

### Correlations Between MT Peptides-Specific Immunoreactivity and Clinical Manifestations

**Figure 3:**
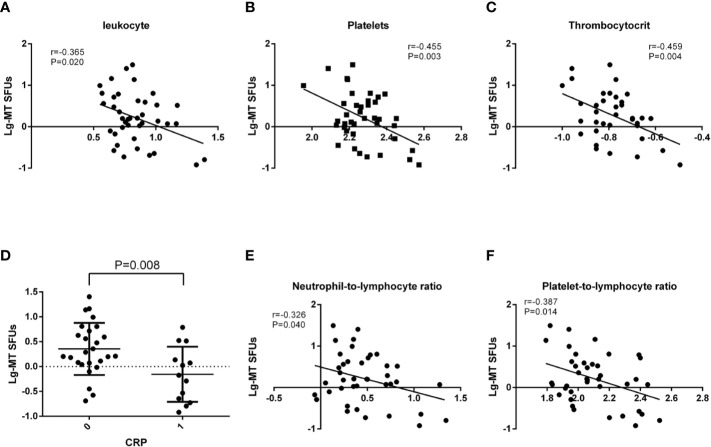
Correlations between MT peptides-specific SFUs and clinical manifestations. **(A–C)** Correlations between the average MT-specific SFUs (MT-SFUs) and the leukocytes **(A)**, platelets **(B)** and thrombocytocrit **(C)**. **(D)** Comparison of the average MT-SFUs between CRP < 8 mg/mL (indicated as “0”) and CRP ≥ 8 mg/mL (indicated as “1”). **(E)** Correlation between the average MT-SFUs and neutrophil-to-lymphocyte ratio. **(F)** Correlation between the average MT-SFUs and platelet-to-lymphocyte ratio. Pearson correlation analysis was used for correlation analysis except for CRP by Student t test.

With an apparent increase in the immunoreactivity to MT peptides in BC patients, we further performed the correlation analysis between clinical laboratory indications and the average IFNγ SFUs specific to MT peptides (MT-SFUs) in 40 BC patients. It was found that there were negative correlations between the average MT-SFUs and the counts of the leukocyte (r = −0.365, P = 0.020, **Figure 3A**), the platelet (r = −0.455, P = 0.003, **Figure 3B**), and thrombocytocrit (r = −0.459, P=0.004, **Figure 3C**). The higher C-reaction protein (CRP) level was, the lower the immunoreactivity to MT peptides displayed (P = 0.008, **Figure 3D**). The average MT-SFUs was negatively correlated with neutrophil proportion, and positively with monocyte proportion and the count of lymphocytes without statistical significance (**Supplementary Figure 4**). Subsequently, neutrophil-to-lymphocyte ratio (NLR) (**Figure 3E**) displayed negative correlation with the average MT-SFUs (r = −0.326, P = 0.040) as well as Platelet-to-lymphocyte ratio (PLR) (r = −0.387, P = 0.014)(**Figure 3F**).

**Figure 4:**
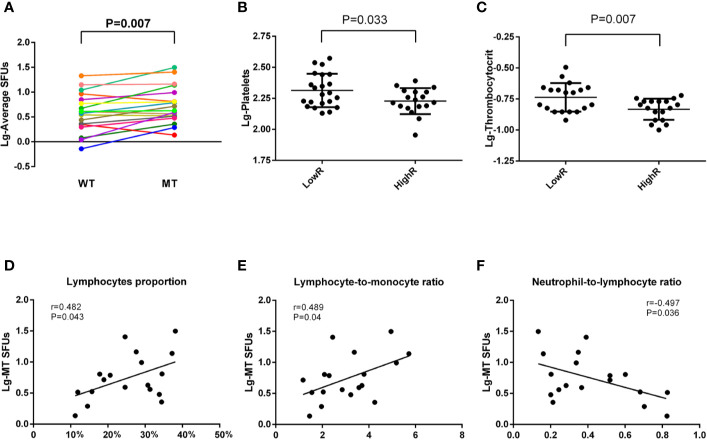
Correlations between MT-SFUs and clinical manifestations in HighR group. Forty HLA-A2^+^ BC patients were subgrouped into LowR (n = 22) and HighR (n = 18) groups according to the SFUs to individual peptides. **(A)** The SFUs upon WT and MT peptides stimulation were compared in HighR group patients. Each line represented one patient in HighR group. **(B, C)** Comparison of the platelet **(B)** and thrombocytocrit **(C)** between LowR and HighR groups. **(D, E)** Correlations between the average MT-SFUs in HighR group and lymphocyte proportion **(D)**, LMR(E) and NLR **(F)**. All data were normally distributed after logarithmic transformation and Pearson correlation analysis was used.

Considering the variety in the immunoreactivity to MT peptides in 40 BC patients, we subgrouped the patients according to the values of MT-SFUs. Eighteen patients (P2-P5, P10, P11, P15-P17, P25, P26, P28, P31-34, P38, and P39) displayed the immunoreactivity with the SFUs more than 5 upon the stimulation of at least two individual MT peptides, which was defined as HighR group. The resting twenty-two patients (P1, P6–P9, P12–P14, P18–P24, P27, P29, P30, P35–37, and P40) were defined as LowR group (**Supplementary Table1**). The average SFUs to MT peptides were higher than to WT peptides in HighR group as well (P=0.007, **Figure 4A**). Consistently, the numbers of the platelet (P = 0.033, **Figure 4B**) and thrombocytocrit (P = 0.007, **Figure 4C**) were lower in HighR group than those in LowR group.

When analyzing the correlations between clinical manifestations and the SFUs to MT peptides in HighR group, it was found that the average MT-SFUs in HighR BC patients exhibited strong positive correlations with the proportion of the lymphocyte (r = 0.482, P = 0.043, **Figure 4D**) and lymphocyte-to-monocyte ratio (LMR, r = 0.489, P = 0.04, **Figure 4E**). Negative correlation with NLR (r = −0.497, P = 0.036, **Figure 4F**) was also present in HighR group (**Supplementary Table 2**).

### Associations Between Hyper-Immunoreactive MT Peptides and Clinical Outcome of BC Patients

MT peptides are predicted from somatic mutations in tumors, which might in turn facilitate anti-tumor immunity and lead to a better survival. Although the disease free survival (DFS) of 40 BC patients was not reachable due to the short period of the observation, we investigated the relationship between somatic mutations of 12 genes and the DFS by using online cBioportal Website resource including 412 BC patients. The DFS in BC patients with or without somatic mutations were plotted and compared. None of a single gene with somatic mutation dedicated to a significantly longer DFS in 412 BC patients. However, BC patients with *CDKN1A, RHOB, DDB1, AHNAK, ANP32A* and *MKI67* genes harboring somatic mutations had relatively longer DFS when compared with unaltered group (**Supplementary Figure 5** and **Supplementary Table 3**).

**Figure 5:**
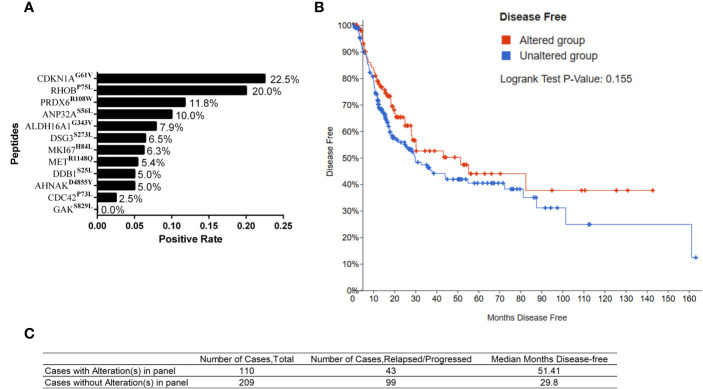
Somatic mutations and clinical outcome from TCGA database. **(A)** Positive rates of the immunoreactivity to individual MT peptides in 40 HLA-A2^+^ BC patients. **(B)** Kaplan–Meier curve of BC patients with (altered group) or without mutations (unaltered group) in 6 genes from the TCGA database. **(C)** The number of the cases and median disease-free months of BC patients with or without mutations in 6 genes.

Considering low frequency of somatic mutations of a single gene in the population, combination of genes with somatic mutations might be more prone in future application. We firstly calculated the positive rate of the immunoreactivity to individual MT peptide in 40 BC patients. Those whose SFUs to a specific MT peptide was more than 5 whereas less than 5 in WT counterpart was defined as positive immunoreactivity. Accordingly, the positive rates of 12 MT peptides in 40 BC patients were calculated. It was found that CDKN1A^G61V^ and RHOB^P75L^ were the two MT peptides with the highest immunoreactivity-positive rates in 40 BC patients (22.5% and 20.0%, respectively) (**Figure 5A**). We therefore chose six genes including *CDKN1A^G61V^, RHOB^P75L^, DDB1^S25L^, AHNAK^D4855Y^, ANP32A^S56L^*, and *MKI67^H84L^* with high immunoreactivity rates in 40 BC patients and possessed relatively longer DFS for combinational analysis. Their immunoreactivity covered 47.5% of the patients under investigation. Patients with one or more somatic mutations in 6 genes exhibited a longer DFS (Median months disease free: 51.41 months) when compared to the cases without mutations (Median months disease free: 29.8 months) although with no significant difference (Log rank Test P value: 0.155, **Figures 5B, C**). Therefore, BC patients harboring 6 hyper-immunoreactive somatic mutations to some extent have a better prognosis in clinic.

## Discussion

Somatic mutations existing in the malignancies can generate neo-”nonself” epitopes and is potentiated in inducing an anti-tumor immunity. Previous studies demonstrated the existence of mutation-specific T cells responses in melanoma (13, 14), gastro-intestinal cancer and lung cancer (8, 19), which facilitates the designing of tumor vaccine as well as peptide-induced adoptive cell therapy in cancer immunotherapy (20, 21). In this study, by using mutation data derived from 412 BC WES data in the TCGA database we have identified 12 MT peptides in BC and demonstrated the existence of mutation-specific immunoreactivity in the periphery of BC patients.

At present the pipeline to define candidate neoantigens from public WES database is well established. In our study, we have filtered MT peptides with both predicted high affinity and expression levels in BC after the comparison to WT counterparts. The top 57 candidate peptide pairs were subjected to the determination of HLA-A2 affinity by a T2 cell-based assay and peptide-specific immunoreactivity in BC patients. Unsurprisingly, the immunoreactivity to MT peptides is dramatically higher than WT peptides. When analyzing the factors associated with mutation-specific immunoreactivity levels in the periphery of BC patients, the most relevant clinical parameters are the counts of leukocytes, platelets and CRP. Both exhibit significantly negative correlations with mutation-specific immunoreactivity. The leukocytes, platelets and CRP have been already reported to be the indications of systemic inflammation (22). CRP is one of soluble pathogen recognition receptors and increased dramatically after infection (23). The platelets and leukocytes serve as the key initiators and participants of the inflammation (24). During the early stage of the infection, the platelets can adhere to the neutrophils and initiate the formation of neutrophil extracellular traps, which in turn activate the platelets. Once activated, the platelets can secrete multiple cytokines and chemokines to exaggerate inflammation which the leukocytes take in charge. Therefore, the negative correlations between mutation-specific immunity and CRP, the platelets/leukocytes imply that inflammation might be one of the decay factors of antigen-specific anti-tumor immunity. This is also consistent with the observations and mechanisms on inflammation-related carcinogenesis (25). Interestingly, somatic mutation-specific immunoreactivity was negatively correlated in more extent with NLR, PLR and LMR in BC patients and high immunoreactive group, which is also the indicators of inflammation (22, 26). Higher NLR is reported to be associated with a poor prognosis during immune checkpoint inhibitor treatment (27, 28), another type of immunotherapy relying on the restore of anti-tumor immunity. These results altogether support that the control of inflammation during cancer immunotherapy is probably benefit for the better outcome of the treatment.

According to our results, the immunoreactivity to somatic mutation-derived peptides is very diverse in BC patients. Fifty-five percent (22/40) of BC patients exhibited rare mutation-specific SFUs while the remaining patients displayed relatively high responses. Although we did not get the information of gene mutant patterns in BC patients investigated, we have surveyed the mutant rates in 412 BC samples by the WES data online. The frequency of mutation rates is very low (from 1–6 cases in 412 BC patients). Nevertheless, mutation-specific immunoreactivity is detectable in most of the HighR patients. We therefore deduce that immune recognition of somatic mutations might be more sensitive than next generation sequencing based detection of somatic mutations in tumor patients. In our study, the immunoreactivity to WT peptides is also observed in certain BC patients with a less extent and one healthy donor have been added to the screening which exhibited few immune-reactivity to peptide stimulation *in vitro*. A recent publication has described the generation of neoantigen-specific T cells from healthy donors (29), which in part support our results that WT-specific T cells might be the evidence on TCR redundancy in BC patients. Another possibility might be due to the overexpression of genes in tumors, which also facilitates the induction of immunoreacitivity in cancer patients. Three genes, including MKI67, DDB1, and ANP32A have been reported to be upregulated in BC already (http://ualcan.path.uab.edu/index.html) (30).

Although most of the studies define mutation-specific immunity in tumor microenvironments (TME) using tumor infiltrating lymphocytes (31), the existence of mutation-specific immunoreactivity in the periphery is still significant. According to a recent report, pre-existing T cells infiltrating in TME were too fragile to be revigorated upon immune checkpoint inhibitor treatment. Peripheral antigen-specific immune cells become the resources that circulate and replace the exhausted T cells inside TME to function as the anti-tumor players (32). MT peptide-specific T cells in the periphery can act as the reservoir of anti-tumor immunity once these mutant peptides are applied as tumor vaccine against BC. In addition, MT peptides might also be subjected to the combination therapy with intravesical BCG administration or other immunotherapy in the future. While intravesical BCG administration induces the infiltration of immune cells inside in a less antigen-specific manner, addition of MT peptides with high-immunogenicity recapitulates the *in vivo* sensitization and orchestrates anti-tumor immunity in an antigen-specific manner.

Taken together, by using public WES data from 412 bladder cancer in the TCGA database we have identified 12 HLA-A*02:01 restricted MT peptides exhibiting stronger immunoreactivity in the periphery of BC patients among which six somatic mutations are partially associated with a better prognosis. MT peptide-specific immunoreactivity is negatively correlated with peripheral inflammatory indicators, providing a potential strategy to enhance the efficacy of neoantigen-based immunotherapy against urothelial carcinoma in the future.

## Data Availability Statement

The original contributions presented in the study are included in the article/**Supplementary Material**. Further inquiries can be directed to the corresponding authors.

## Ethics Statement

The studies involving human participants were reviewed and approved by Medical Ethics Committee of Xinhua Hospital affiliated to Shanghai Jiaotong University School of Medicine. The patients/participants provided their written informed consent to participate in this study.

## Author Contributions

YW and HS conceived the project and designed the experiments. CW, YD, and YL performed the experiments. QZ and ZC performed the bioinformatics analysis. CW, YD, SX, and HD collected the blood samples and clinical data. LX, SW, and PJ provided technique support. GZ, YW, and WH initiated the projects. YW, HS, and CW performed data analysis. YW, HS, ZC, CW, YD, and QZ drafted the manuscript. YW and HS were senior authors. All authors contributed to the article and approved the submitted version.

## Funding

This work was supported by the grant from National Key R&D Program of China (2016YEC1303300) and Shanghai Academic Research Leader Project (2018XD1403300).

## Conflict of Interest

The authors declare that the research was conducted in the absence of any commercial or financial relationships that could be construed as a potential conflict of interest.
