# Supplementary material for: Identification of Mutated Peptides in Bladder Cancer From Exomic Sequencing Data Reveals Negative Correlation Between Mutation-Specific Immunoreactivity and Inflammation
Source: Front Immunol. 2020 Nov 30;11:576603. doi: 10.3389/fimmu.2020.576603 (PMC7734250; doi:10.3389/fimmu.2020.576603)
Supplement: Supplementary file 1 [file DataSheet_1.docx]

**Identification of mutated peptides in bladder cancer from exomic sequencing data reveals negative correlation between mutation-specific immunoreactivity and inflammation**

**Chen Wang^1*^, Yu Ding^1*^, Yuanyong Liu^2*^, Qingchen Zhang^3^, Shiqiang Xu^2^, Liliang Xia^2^, Huangqi Duan^1^, Shujun Wang^2^, Ping Ji^2^, Weiren Huang^4^, Guoping Zhao^5^, Zhiwei Cao^3^, Haibo Shen^1^, Ying Wang^2,5^**

**# Correspondence:**Prof. Ying Wang

Shanghai Institute of Immunology, Department of Immunology and Microbiology, Shanghai Jiaotong University School of Medicine, Shanghai 200025, China

Email: ywang@sibs.ac.cn

And

Dr. Hai-bo Shen

Department of Urology, Xinhua Hospital, Shanghai Jiaotong University School of Medicine, Shanghai, 200092, China

Email: shenhaibo@xinhuamed.com.cn

## Supplementary Figures


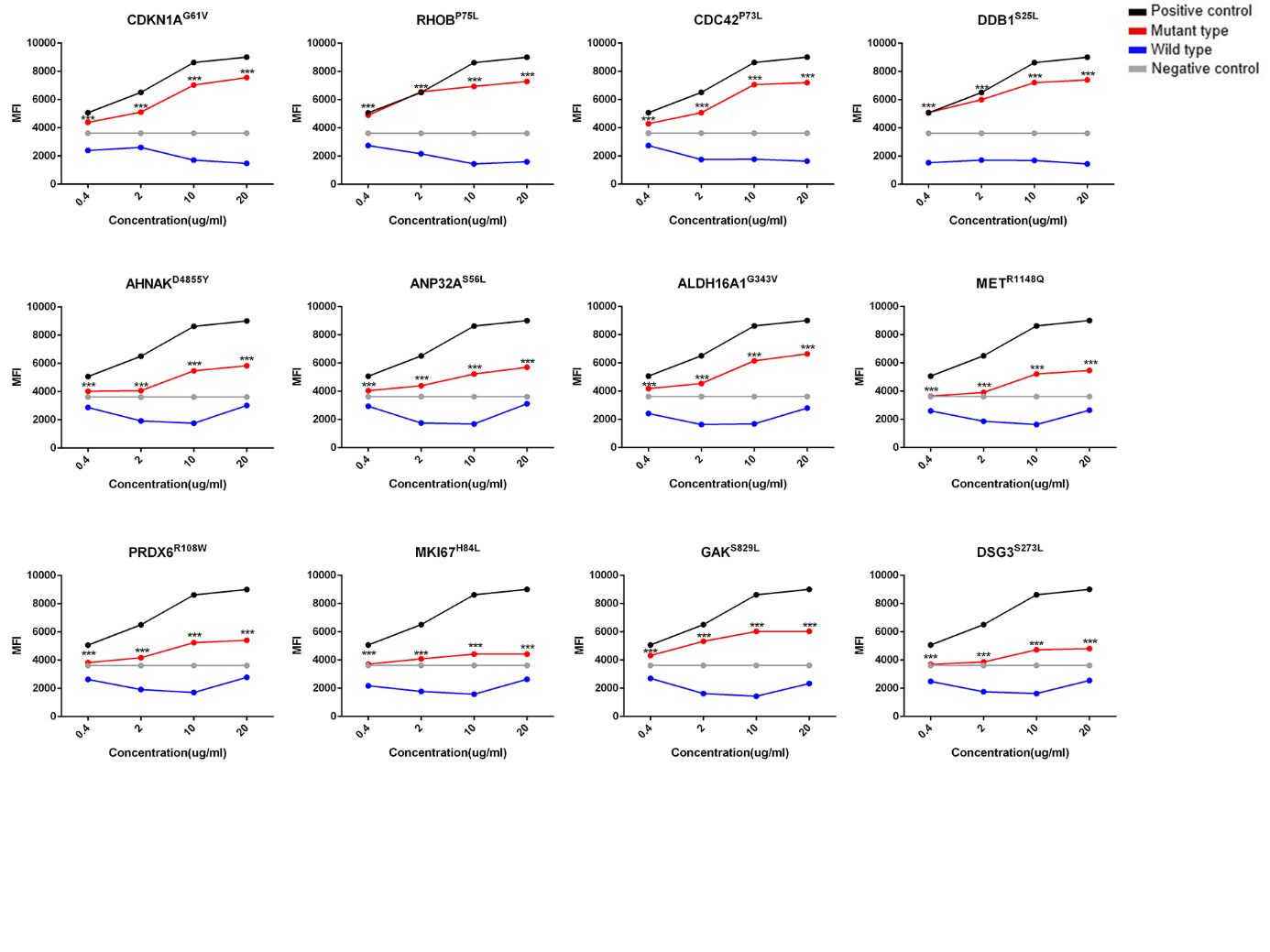


**Supplementary Figure1.** Binding affinity assays on wild type and mutant peptides at different concentrations (***: P<0.001).





**Supplementary Figure2.** Periphery immunoreactivity to WT and MT peptide in one HLA-A2(+) health donor.


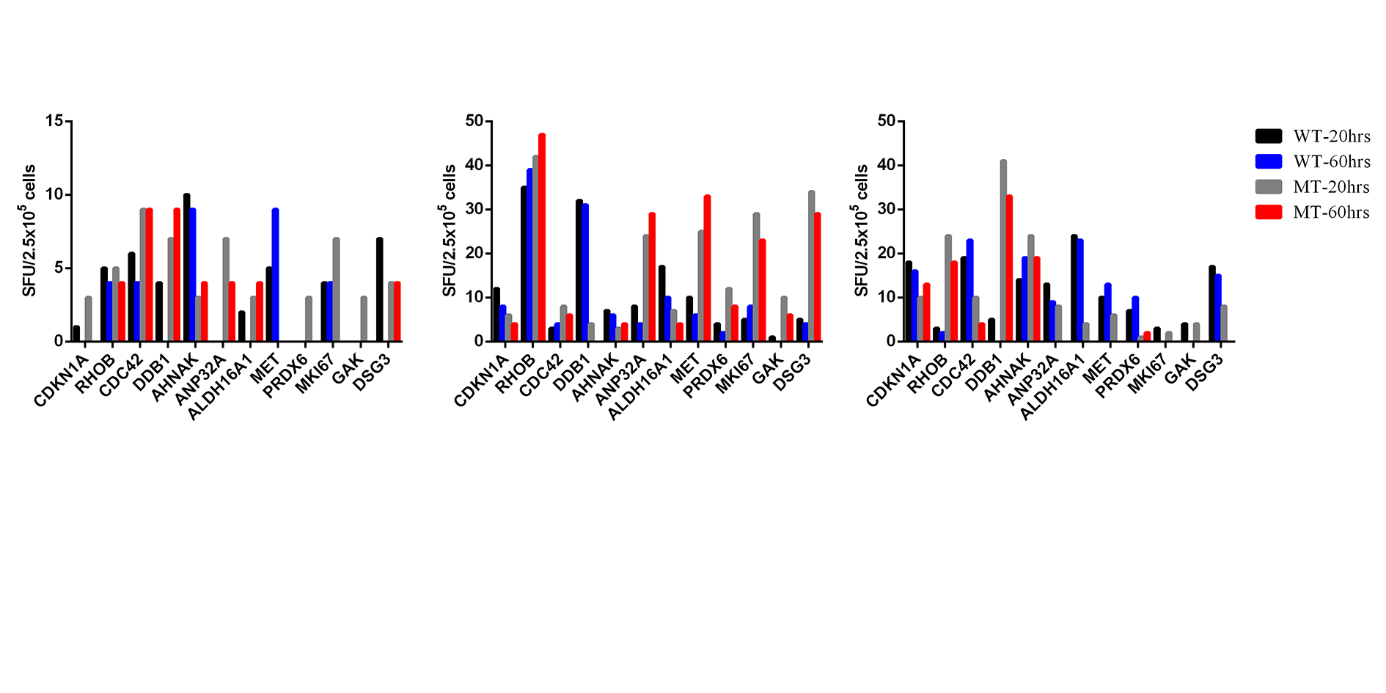


**Supplementary Figure3.** Comparison of IFN-γ releases upon stimulation of WT and MT peptides between 20 hrs and 60 hrs in 3 HLA-A2(+) BC patients by an ELISPOT assay.


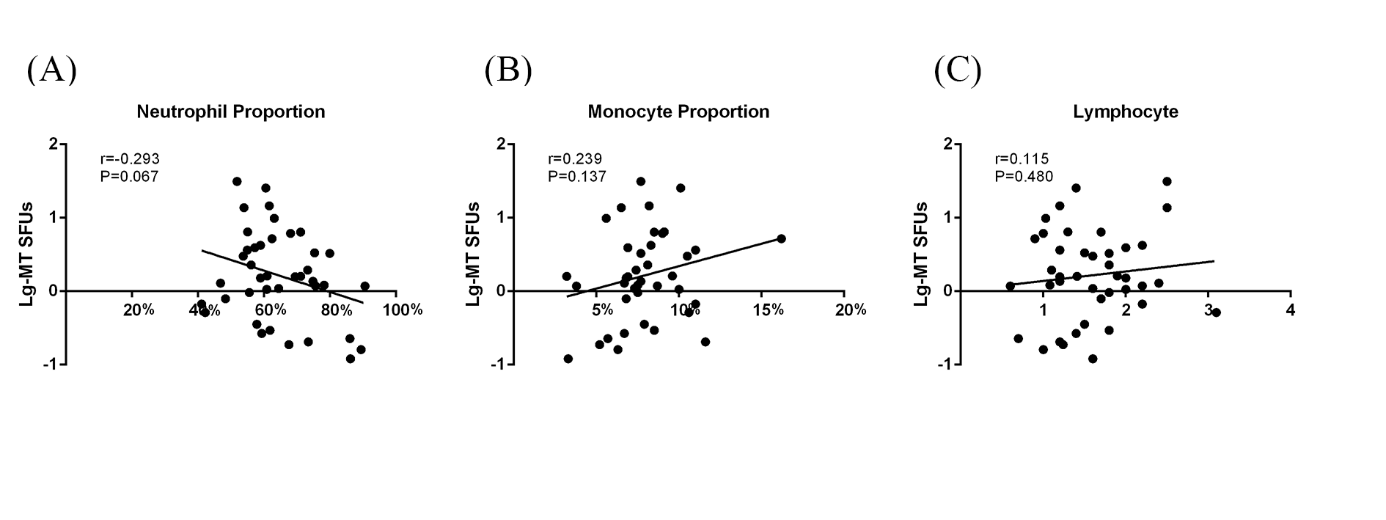
**Supplementary Figure4.** Correlations between MT peptides-specific SFUs and clinical manifestations

All data were normally distributed after logarithmic transformation. Pearson correlation analyses were performed between the average MT-SFUs and neutrophil proportion (A), monocyte proportion (B) and lymphocyte (C), P > 0.05.


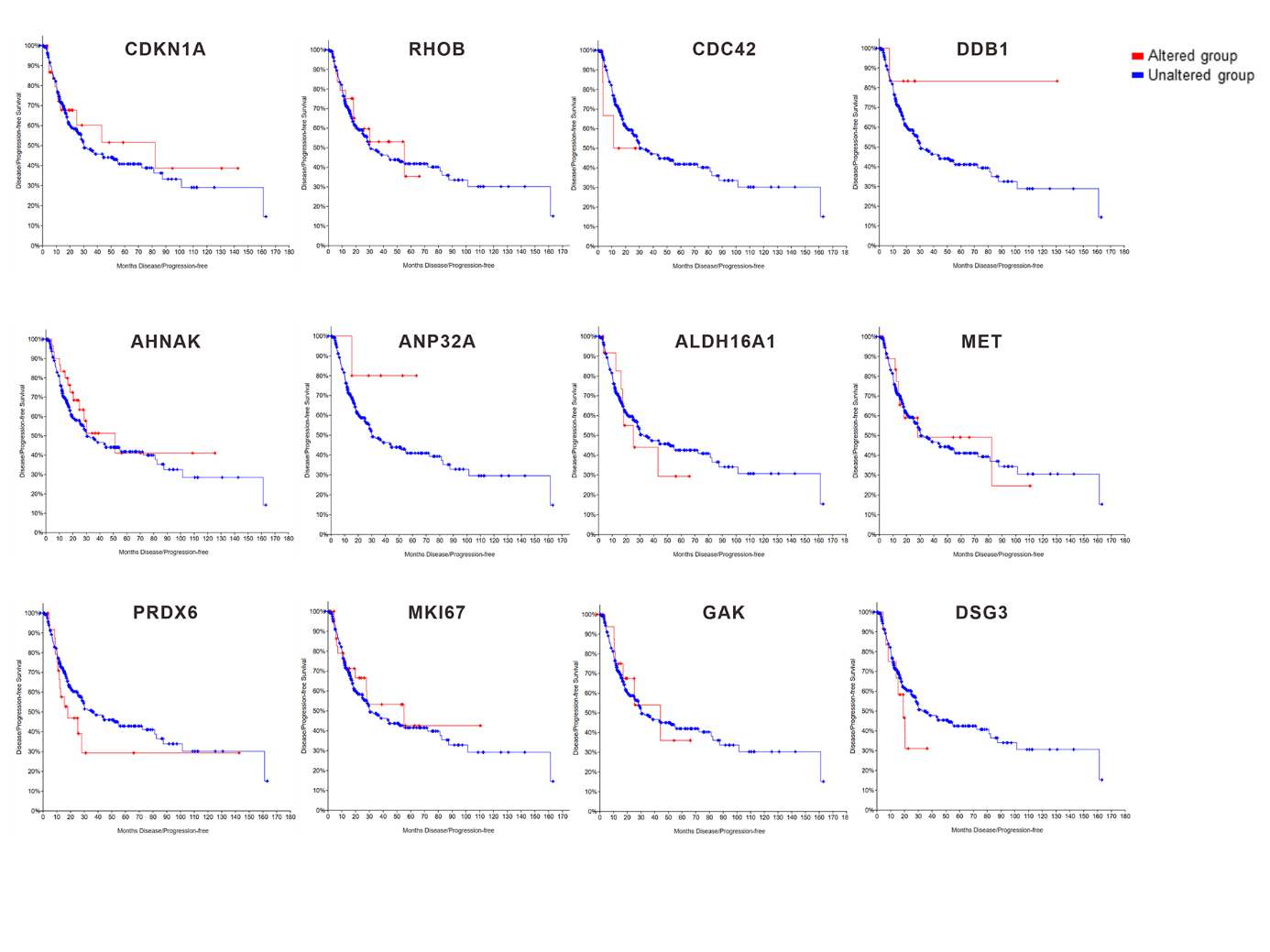


**Supplementary Figure5.** K-M plot of the DFS curves of 12 genes with or without alterations according to in silicon cBio Cancer Genomics Portal analysis.

**Supplementary Table1.** Summary of peptide-specific IFNγ spot forming units in 40 BC patients

| No |  | CDKN1A^G61V^ | RHOB^P75L^ | CDC42^P73L^ | DDB1^S25L^ | AHNAK^D4855Y^ | ANP32A^S56L^ | ALDH16A1^G343V^ | MET^R1148Q^ | PRDX6^R108W^ | MKI67^H84L^ | GAK^S829L^ | DSG3^S273L^ |
| --- | --- | --- | --- | --- | --- | --- | --- | --- | --- | --- | --- | --- | --- |
| P1 | WT | 0 | 0 | 1 | 0 | 0 | 0 | 4 | 0 | 4 | 0 | 0 | 2 |
|  | MT | 2 | 2 | 0 | 1 | 4 | 4 | 1 | 0 | 0 | 0 | 1 | 1 |
| P2 | WT | 4 | 5 | 4 | 7 | 4 | 2 | 5 | 7 | 5 | 5 | 0 | 2 |
|  | MT | 5 | 7 | 4 | 7 | 5 | 2 | 6 | 5 | 5 | 5 | 0 | 2 |
| P3 | WT | 0 | 0 | 0 | 6 | 5 | 6 | 6 | 0 | 10 | 0 | 0 | 0 |
|  | MT | 1 | 4 | 1 | 11 | 7 | 9 | 7 | 2 | 12 | 8 | 0 | 0 |
| P4 | WT | 3 | 15 | 17 | 12 | 13 | 24 | ND | ND | ND | ND | ND | ND |
|  | MT | 21 | 16 | 15 | 22 | 6 | 7 | ND | ND | ND | ND | ND | ND |
| P5 | WT | 38 | 38 | 15 | 18 | 13 | 6 | 24 | 24 | 16 | ND | ND | ND |
|  | MT | 45 | 37 | 37 | 26 | 4 | 0 | 11 | 20 | 50 | ND | ND | ND |
| P6 | WT | 0 | 2 | 0 | 2 | 1 | 0 | 5 | 0 | 1 | 0 | 0 | 0 |
|  | MT | 3 | 0 | 3 | 1 | 1 | 1 | 0 | 0 | 0 | 0 | 2 | 0 |
| P7 | WT | 0 | 0 | 0 | 0 | 0 | 0 | ND | 1 | 0 | 0 | 2 | ND |
|  | MT | 2 | 0 | 3 | 0 | 0 | 1 | ND | 2 | 0 | 6 | 1 | ND |
| P8 | WT | 0 | 0 | 0 | 0 | 1 | 6 | 0 | 0 | 1 | 1 | 0 | 0 |
|  | MT | 0 | 0 | 0 | 4 | 2 | 0 | 0 | 0 | 12 | 0 | 0 | 0 |
| P9 | WT | 0 | 1 | 0 | 2 | 1 | 0 | 1 | 1 | 2 | 1 | 0 | 0 |
|  | MT | 1 | 1 | 0 | 0 | 0 | 0 | 0 | 3 | 0 | 0 | 1 | 0 |
| P10 | WT | 3 | 1 | 8 | 2 | 13 | 8 | 6 | ND | ND | ND | ND | ND |
|  | MT | 13 | 1 | 4 | 2 | 4 | 1 | 18 | ND | ND | ND | ND | ND |
| P11 | WT | 22 | 4 | 0 | 0 | 4 | 0 | 9 | 13 | 4 | 0 | 0 | 0 |
|  | MT | 4 | 0 | 26 | 0 | 0 | 31 | 31 | 13 | 57 | 0 | 4 | 0 |
| P12 | WT | 0 | 0 | 0 | 1 | 1 | 0 | 1 | 0 | 1 | 0 | 1 | 0 |
|  | MT | 2 | 1 | 1 | 1 | 1 | 1 | 1 | 2 | 1 | 0 | 2 | 1 |
| P13 | WT | 0 | 0 | 2 | 1 | 1 | 0 | 2 | 0 | 0 | 1 | 1 | 2 |
|  | MT | 5 | 0 | 2 | 2 | 0 | 3 | 0 | 1 | 0 | 0 | 0 | 0 |
| P14 | WT | 0 | 0 | 0 | 11 | 2 | 1 | 0 | 15 | 0 | 0 | 3 | 1 |
|  | MT | 2 | 15 | 0 | 0 | 0 | 0 | 2 | 0 | 0 | 0 | 0 | 0 |
| P15 | WT | 4 | 5 | 9 | 2 | 26 | 18 | 4 | 20 | ND | ND | ND | ND |
|  | MT | 10 | 79 | 0 | 73 | 59 | 1 | 29 | 1 | ND | ND | ND | ND |
| P16 | WT | 3 | 0 | 0 | 2 | 1 | 10 | 7 | 7 | ND | ND | ND | ND |
|  | MT | 7 | 11 | 2 | 3 | 1 | 15 | 6 | 4 | ND | ND | ND | ND |
| P17 | WT | 1 | 0 | 0 | 1 | 1 | 1 | 1 | 0 | ND | ND | ND | ND |
|  | MT | 2 | 2 | 2 | 0 | 0 | 1 | 5 | 5 | ND | ND | ND | ND |
| P18 | WT | 0 | 0 | 1 | 0 | 0 | 0 | 0 | 1 | 0 | 0 | 0 | 0 |
|  | MT | 0 | 2 | 0 | 0 | 0 | 0 | 1 | 0 | 0 | 0 | 1 | 0 |
| P19 | WT | 0 | 2 | 1 | 0 | 0 | 1 | 0 | 0 | 0 | 0 | 1 | 1 |
|  | MT | 0 | 12 | 0 | 0 | 0 | 2 | 0 | 1 | 0 | 0 | 2 | 3 |
| P20 | WT | 0 | 0 | 0 | 0 | 0 | 0 | 0 | 0 | ND | ND | ND | ND |
|  | MT | 0 | 1 | 0 | 0 | 0 | 0 | 0 | 0 | ND | ND | ND | ND |
| P21 | WT | 1 | 1 | 0 | 1 | 1 | 0 | 1 | 0 | 0 | 0 | 0 | 0 |
|  | MT | 0 | 0 | 0 | 0 | 0 | 1 | 1 | 0 | 0 | 0 | 1 | 0 |
| P22 | WT | 1 | 0 | 0 | 0 | 0 | 0 | 0 | 0 | 0 | 1 | 0 | 0 |
|  | MT | 0 | 0 | 0 | 0 | 0 | 1 | 0 | 0 | 1 | 0 | 0 | 0 |
| P23 | WT | 0 | 0 | 0 | 0 | 1 | 0 | 0 | 0 | 0 | 0 | 0 | 1 |
|  | MT | 0 | 0 | 0 | 0 | 1 | 0 | 0 | 1 | 0 | 1 | 0 | 0 |
| P24 | WT | 1 | 1 | 1 | 1 | 0 | 1 | 0 | 1 | 6 | 1 | 0 | 1 |
|  | MT | 2 | 4 | 0 | 2 | 1 | 1 | 0 | 1 | 1 | 0 | 0 | 2 |
| P25 | WT | 1 | 2 | 0 | 1 | 5 | 0 | 2 | 0 | 0 | 1 | 2 | 1 |
|  | MT | 0 | 11 | 0 | 1 | 1 | 5 | 1 | 0 | 5 | 2 | 0 | 21 |
| P26 | WT | 0 | 5 | 6 | 7 | 15 | 8 | 7 | 6 | 7 | 9 | 10 | 5 |
|  | MT | 9 | 26 | 10 | 9 | 10 | 10 | 3 | 11 | 8 | 8 | 10 | 4 |
| P27 | WT | 1 | 0 | 1 | 0 | 0 | 0 | 2 | 1 | 0 | 0 | 0 | 2 |
|  | MT | 1 | 2 | 2 | 1 | 0 | 0 | 0 | 0 | 0 | 1 | 2 | 1 |
| P28 | WT | 0 | 0 | 7 | 8 | 0 | 0 | 0 | 4 | 5 | 0 | 0 | 0 |
|  | MT | 11 | 12 | 0 | 4 | 0 | 2 | 1 | 6 | 0 | 0 | 0 | 0 |
| P29 | WT | 1 | 0 | 0 | 3 | 0 | 0 | 0 | 1 | 2 | 2 | 2 | 0 |
|  | MT | 0 | 0 | 0 | 0 | 0 | 0 | 0 | 1 | 0 | 0 | 0 | 0 |
| P30 | WT | 0 | 2 | 1 | 4 | 0 | 2 | 1 | 2 | 1 | 1 | 2 | 1 |
|  | MT | 3 | 1 | 1 | 1 | 2 | 1 | 1 | 4 | 1 | 1 | 1 | 1 |
| P31 | WT | 0 | 4 | 0 | 0 | 0 | 0 | 8 | 7 | 8 | 0 | 0 | 1 |
|  | MT | 0 | 14 | 1 | 5 | 0 | 3 | 5 | 4 | 7 | 0 | 0 | 0 |
| P32 | WT | 2 | 0 | 5 | 9 | 0 | 0 | 2 | 5 | 0 | 9 | 12 | 5 |
|  | MT | 0 | 14 | 2 | 2 | 3 | 0 | 12 | 2 | 5 | 0 | 0 | 3 |
| P33 | WT | 2 | 0 | 0 | 16 | 0 | 0 | 3 | 0 | 0 | 5 | 0 | 0 |
|  | MT | 0 | 3 | 1 | 5 | 6 | 0 | 0 | 0 | 0 | 0 | 0 | 0 |
| P34 | WT | 9 | 7 | 12 | 6 | 8 | 13 | 10 | 7 | 12 | 8 | 6 | 11 |
|  | MT | 3 | 2 | 9 | 9 | 12 | 5 | 5 | 7 | 5 | 12 | 3 | 5 |
| P35 | WT | 1 | 1 | 0 | 0 | 4 | 2 | 0 | 0 | 2 | 0 | 1 | 0 |
|  | MT | 4 | 3 | 0 | 0 | 0 | 2 | 0 | 2 | 2 | 0 | 0 | 2 |
| P36 | WT | 0 | 0 | 0 | 0 | 0 | 0 | 0 | 1 | 0 | 0 | 0 | 0 |
|  | MT | 0 | 0 | 0 | 0 | 0 | 0 | 0 | 2 | 0 | 0 | 0 | 0 |
| P37 | WT | 0 | 0 | 0 | 0 | 0 | 0 | 2 | 0 | 0 | ND | ND | ND |
|  | MT | 0 | 0 | 0 | 1 | 0 | 2 | 2 | 1 | 0 | ND | ND | ND |
| P38 | WT | 0 | 2 | 6 | 0 | 13 | 2 | 7 | 6 | 0 | 0 | 5 | 0 |
|  | MT | 4 | 5 | 6 | 3 | 0 | 6 | 0 | 0 | 0 | 2 | 4 | 10 |
| P39 | WT | 0 | 1 | 5 | 0 | 0 | 0 | 3 | 0 | 0 | 4 | 1 | 0 |
|  | MT | 5 | 0 | 0 | 4 | 2 | 5 | 0 | 3 | 0 | 1 | 4 | 3 |
| P40 | WT | 0 | 0 | 0 | 0 | 0 | 0 | 0 | 4 | 0 | 0 | 0 | 2 |
|  | MT | 0 | 0 | 0 | 0 | 0 | 0 | 0 | 0 | 0 | 2 | 0 | 0 |

ND: not determined

**Supplementary Table2.** The correlations between clinical manifestations and the average SFUs induced by mutant peptides

| **clinical information** | **r** | **P value** |
| --- | --- | --- |
| leukocyte | -0.365 | 0.020 |
| platelet | -0.455 | 0.003 |
| thrombocytocrit | -0.459 | 0.004 |
| NLR | -0.326 | 0.040 |
| PLR | -0.387 | 0.014 |
| neutrophil proportion | -0.293 | 0.067 |
| monocyte proportion | 0.239 | 0.137 |
| lymphocyte | 0.115 | 0.480 |
| **HighR group** |  |  |
| lymphocyte proportion | 0.482 | 0.043 |
| LMR | 0.489 | 0.040 |
| NLR | -0.497 | 0.036 |

**Supplementary Table3.** Summary of P values of the DFS of bladder cancer patients with or without mutations in twelve individual genes

| Gene | Cases | Total | Relapsed/Progressed | Median Months Disease-free | P-Value |
| --- | --- | --- | --- | --- | --- |
| CDKN1A | With Alteration(s) | 32 | 12 | 82.42 | 0.62 |
|  | Without Alteration(s) | 287 | 130 | 30.12 |  |
| RHOB | With Alteration(s) | 26 | 11 | 55.16 | 0.691 |
|  | Without Alteration(s) | 293 | 131 | 30.22 |  |
| CDC42 | With Alteration(s) | 7 | 3 | 11.27 | 0.4 |
|  | Without Alteration(s) | 312 | 139 | 30.22 |  |
| DDB1 | With Alteration(s) | 7 | 1 | NA | 0.183 |
|  | Without Alteration(s) | 312 | 141 | 30.12 |  |
| AHNAK | With Alteration(s) | 32 | 13 | 51.41 | 0.367 |
|  | Without Alteration(s) | 287 | 129 | 30.12 |  |
| ANP32A | With Alteration(s) | 5 | 1 | NA | 0.193 |
|  | Without Alteration(s) | 314 | 141 | 30.12 |  |
| ALDH16A1 | With Alteration(s) | 13 | 7 | 25.23 | 0.629 |
|  | Without Alteration(s) | 306 | 135 | 32.59 |  |
| MET | With Alteration(s) | 20 | 9 | 27.99 | 0.868 |
|  | Without Alteration(s) | 299 | 133 | 30.22 |  |
| PRDX6 | With Alteration(s) | 27 | 14 | 18 | 0.187 |
|  | Without Alteration(s) | 292 | 128 | 36.86 |  |
| MKI67 | With Alteration(s) | 33 | 12 | 55.16 | 0.555 |
|  | Without Alteration(s) | 286 | 130 | 30.22 |  |
| GAK | With Alteration(s) | 18 | 7 | 44.15 | 0.83 |
|  | Without Alteration(s) | 301 | 135 | 30.22 |  |
| DSG3 | With Alteration(s) | 12 | 7 | 19.09 | 0.28 |
|  | Without Alteration(s) | 307 | 135 | 32.59 |  |

**Supplementary Table 3** The correlations between clinical manifestations and the average SFUs induced by mutant peptides

| **clinical information** | **r** | **P value** |
| --- | --- | --- |
| leukocyte | -0.365 | 0.020 |
| platelet | -0.455 | 0.003 |
| thrombocytocrit | -0.459 | 0.004 |
| NLR | -0.326 | 0.040 |
| PLR | -0.387 | 0.014 |
| neutrophil proportion | -0.293 | 0.067 |
| monocyte proportion | 0.239 | 0.137 |
| lymphocyte | 0.115 | 0.480 |
| **HighR group** |  |  |
| lymphocyte proportion | 0.482 | 0.043 |
| LMR | 0.489 | 0.040 |
| NLR | -0.497 | 0.036 |
